# A Peculiar Case of Fentanyl-Induced Cardiomyopathy

**DOI:** 10.7759/cureus.27708

**Published:** 2022-08-05

**Authors:** Faraz Badar, Aqsa Ashraf, Md R Bhuiyan, Tia Bimal, Asma Iftikhar

**Affiliations:** 1 Internal Medicine, Mather Hospital at Northwell Health, Port Jefferson, USA; 2 Pulmonary and Critical Care Medicine, Mather Hospital at Northwell Health, Port Jefferson, USA

**Keywords:** rare side effect, fentanyl analogs, cardiac arrythmia, opioid use disorders, cardio toxicity

## Abstract

An alarming rise in prescription and non-prescription misuse of opioids has been observed recently, leading to potentially devastating consequences. Opioid misuse contributes to cardiac risk burden and can cause diseases such as acute coronary syndrome, congestive heart failure, arrhythmias, QTc prolongation, and endocarditis. Here, we describe the case of a 35-year-old male with recreational fentanyl use who was found to have a cardiogenic shock on point-of-care ultrasound (POCUS), likely due to fentanyl-induced cardiomyopathy. Opioid-induced cardiomyopathy without any underlying cardiac disease in an adult appears to be a rare case. Our case highlights the importance of promptly recognizing fentanyl toxicity, screening for possible cardiomyopathy secondary to its use, and emergent resuscitation with the maintenance of ventilation, diuretics, and vasopressor support. The use of the reversal agent, naloxone, is a crucial part of management.

## Introduction

Opioids are a class of substances that are used vastly in medicine for acute pain, anesthesia, and chronic non-cancer pain. They have a myriad of effects such as analgesia, sedation, respiratory depression, euphoria, miosis, constipation, pruritus, dependence, and tolerance [1,2]. Recently, there has been an alarming rise in both prescription and illicit use of these controlled substances. Highly addictive potential has led to an increase in fatal side effects and overdose-related deaths [1-4].

Cardiovascular medicine has also been affected by this opioid epidemic and a rise in cardiac diseases such as acute coronary syndrome, congestive heart failure, arrhythmias, QTc prolongation, and endocarditis is being associated with opioid misuse or overdose [5-8]. Along these lines, we report a peculiar case of cardiomyopathy caused by illicit fentanyl use, a drug that does not appear to be a commonly reported cause of drug-induced cardiomyopathy in adults. We came across one pediatric case report mentioning a very similar case [9].

## Case presentation

A 35-year-old male presented to our hospital’s emergency department after being found unresponsive in bed by his father. He was reported to be last seen normal at 10 pm the night prior. Emergency medical services administered 2mg of Narcan® with resultant awakening. At bedside, the patient was lethargic but awake, oriented, and conversant. He voiced no acute complaints and denied any chest pain, palpitations, or shortness of breath. The patient endorsed nonprescription fentanyl use the previous night, both via inhalation and oral routes. He denied any other kind of recent substance use.

Past medical history was notably positive for psoriasis treated with adalimumab infusions, chronic back pain, and opioid use disorder with oxycodone for which he underwent rehabilitation. Social history was positive for current cigarette smoking with eight pack years (0.5 packs per day for 15 years) and weekly consumption of three to four alcoholic beverages. The patient denied having multiple sexual partners, lived with his father and two sons, and was self-employed. 

Initial vital signs were temperature of 97.5^o^F, tachycardia of 126 beats per minute, tachypnea of 28 breaths per minute, oxygen saturation of 85% on room air, and hypotension to 67/48 mmHg (calculated mean arterial pressure (MAP) 54). On physical examination, the patient was drowsy but arousable, alert, and oriented to time, place, and person. Pupils were reactive and constricted to 3mm equally. Skin findings included multiple tattoos and erythematous patches with silvery scales on bilateral knees consistent with psoriasis. Chest auscultation had regular tachycardia with no murmurs whereas lung auscultation had clear air entry bilaterally with no appreciable crackles or wheezing. Abdomen was non-tender and non-distended. Lower extremity edema was absent. Laboratory test results are given in Table 1.

**Table 1:** Initial laboratory test results WBC: white blood count; BUN: blood urea nitrogen; pro-BNP: N-terminal prohormone of brain natriuretic peptide; TSH: thyroid-stimulating hormone; CPK: creatinine phosphokinase; pCO2: partial pressure of carbon dioxide; pO2: partial pressure of oxygen; FiO2: fraction of inspired oxygen

Hematology		
Name	Result	Reference Range
WBC	6.8	3.5 - 10.8 K/ul
Hemoglobin	14.5	11.5 - 15.5 g/dl
Hematocrit	42.4	34.5 - 45.0 %
Platelet Count	214	150 - 400 K/ul
General Chemistry		
Name	Result	Reference Range
Sodium	135	136 - 145 mmol/L
Potassium	4.0	3.3 - 5.1 mmol/L
Chloride	99	98 - 107 mmol/L
Bicarbonate	33	22 - 29 mmol/L
BUN	9	8 - 23 mg/dl
Creatinine	1.0	0.7 - 1.2 mg/dl
Glucose	110	74 - 109 mg/dl
Magnesium	1.3	1.6 - 2.6 mg/dl
Phosphorus	3.5	2.5 - 4.5 mg/dl
pro-BNP	347	1 - 125 pg/ml
TSH	0.500	0.27 - 4.2 uIU/ml
Cardiac Enzymes		
Name	Result	Reference Range
Troponin T	59 → 57 → 46	<14 ng/L
CPK	8279	120 - 180 U/L
Arterial Blood Gas		
pH	7.25	7.38 - 7.46
pCO2	41	32 - 46 mmHg
pO2	160	74 - 108 mmHg
O2 saturation	99	92 - 96 %
FiO2	100	21 - 100 %
Urine Toxicology		
Amphetamine metabolites	Negative	Negative
Barbiturates	Negative	Negative
Benzodiazepines	Negative	Negative
Cannabinoids	Negative	Negative
Cocaine	Negative	Negative
Opioids	Positive	Negative
Fentanyl metabolite	292	<10 ng/mL
Phencyclidine	Negative	Negative

**Figure 1:**
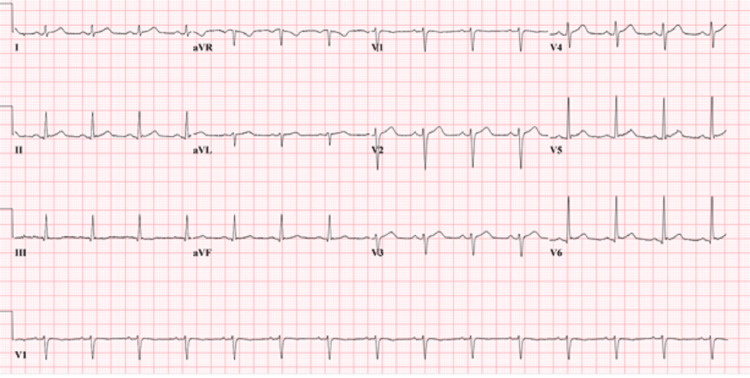
Unremarkable EKG demonstrating normal sinus rhythm and no ST-T wave abnormalities. QTc: 423ms

EKG showed normal sinus rhythm, QTc of 423ms, and no ST-T wave abnormalities ( Figure 1).

Chest x-ray showed bilateral infiltrates involving both right and left peri-hilar regions and lower lobes. No cardiomegaly was appreciated. Bedside POCUS was notably positive for B-line pattern, severe global left ventricular dysfunction, ejection fraction (EF) was 15-20%, and left ventricular outflow tract velocity time integral (LVOT VTI) was 7.7cm.

The patient was, therefore, admitted to ICU for cardiogenic shock possibly from fentanyl-induced cardiomyopathy and acute hypoxic respiratory failure from cardiogenic pulmonary edema requiring noninvasive positive pressure ventilation. Emergent resuscitation included noninvasive positive pressure ventilation, administration of furosemide 20mg intravenous push, and norepinephrine vasopressor peripherally. However, hypotension was refractory despite maximal dosing of 30 mcg/min. Therefore, central venous catheter and arterial line were inserted and dobutamine was started for inotropic support at 5 µg/kg/min with a brisk response in MAP to greater than 65. Transthoracic echocardiography the next day revealed mild global hypokinesis of the left ventricle and right wall motion abnormality. EF recovered to 40-45%. The LV wall thickness was normal, diastolic function was indeterminate and there was no valvular abnormality. Due to wall motion abnormalities and mild hypokinesis, cardiac catheterization via right radial artery was performed, which revealed right dominant anatomy with patent and normal coronary arteries. Left ventriculography showed apical tardokinesis and EF again at 40-45%. Vasopressors and noninvasive ventilation were successfully weaned off shortly after.

The patient was started on metoprolol succinate 25 mg daily and losartan 25 mg daily, counseled on drug and tobacco cessation, advised to follow up with cardiology within one week and obtain repeat echocardiography in eight weeks to reassess EF.

## Discussion

Opioids are a common class of drugs in medicine that are used for a vast number of indications such as acute pain, anesthesia, chronic non-cancer pain, cough, diarrhea, and shortness of breath. These drugs act on the μ, κ, and δ opioid receptors found mainly in the central nervous system, peripheral nervous system, and gastrointestinal tract and induce a myriad of effects such as analgesia, sedation, respiratory depression, euphoria, miosis, constipation, pruritus, dependence, tolerance, etc.

Recently, there has been an alarming rise in both prescription and nonprescription use of these controlled substances. Highly addictive potential and fatal side effects have led to an increase in opioid-related overdose deaths; 68,000 deaths were reported in the US in 2020, which was 8.5 times the number of deaths in 1999 [1-4]. More than 70% of deaths occurred among males. People in the age group of 35-44 years and of non-Hispanic American Indian and Alaska Native ethnicity had the highest death rates. Understandably, cardiovascular medicine has also been affected by this opioid epidemic and a rise in cardiovascular diseases such as acute coronary syndrome, congestive heart failure, arrhythmias, QTc prolongation, and endocarditis is being associated with opioid overdose [5-8].

Mechanisms behind cardiovascular effects of opioid receptor agonism include bradycardia and vasodilation resulting in hypotension, orthostasis, syncope, and peripheral edema [2,8,10]. Conduction abnormalities stem from bradycardia as well as sodium, potassium, and calcium channel blockade [2,6,8,11,12,13]. The combination of these vascular and contractility anomalies leads to acute MI, arrhythmias, and other well-known complications of opioid misuse such as sudden death and cardiac and respiratory arrest [5,6,8]. Fentanyl is a synthetic opioid available most commonly in intravenous and transdermal formulations for analgesia, anesthesia, and sedation. Nowadays, it is also used as a recreational drug, mixed with other substances, for example, heroin, cocaine, benzodiazepines, or methamphetamine [1-4,14-16].

Our case is unique as it reports a rare occurrence of drug-induced cardiomyopathy from this opioid in the absence of any underlying cardiac disease. Fentanyl is well known for overdose deaths, usually from cardiopulmonary arrest when it is admixed with other drugs mentioned above [14-16]. As per our literature review, it does not appear to be a commonly reported direct cause of cardiomyopathy or acute onset heart failure with reduced EF (HFrEF) in adults. A pediatric case report with a similar presentation has been published in 2017 [9]. A thorough toxicology workup in our patient did not reveal co-ingestion of any other substance along with the fentanyl. 

A brief summary of the commonly reported drugs associated with direct cardiomyotoxicity is given in Table 2.

**Table 2:** Commonly reported drugs associated with direct cardiomyotoxicity

Drug Class	Common example	Reference
Antimalarial	Chloroquine	Yogasundaram et al. [17]
Antiretroviral	Zidovudine	d'Amati et al. [18]
Antipsychotic	Clozapine	Rostagno et al. [19]
Anthracyclines	Doxorubicin	Steinherz et al. [20]
Monoclonal antibodies	Trastuzumab	Keefe et al. [21]
Tyrosine kinase inhibitors	Imatinib	Kerkelä et al. [22]
Alkylating agents	Cyclophosphamide	Gottdiener et al. [23]
Alcohol and cocaine are well-established causes of cardiomyopathy as well		

Usually, fentanyl or opioid overdose is a clinical diagnosis requiring prompt assessment and management. Common clinical features include altered mental status ranging from lethargy to coma, miosis, bradycardia, hypotension, and hypoxia. A unique feature specific to fentanyl is “wooden chest syndrome”, which involves rigidity of the abdominal muscles and diaphragm, inducing respiratory failure [24]. 

Diagnostic modalities include finger stick glucose check to rule out hypoglycemia, serum creatine phosphokinase (CPK) to rule out rhabdomyolysis from immobilization, Chest x-ray to check for possible aspiration, urine/serum toxicology screening, and EKG to assess QTc, especially in cases of a methadone overdose. Management centers on ensuring adequate ventilation via oxygen supplementation in case of spontaneous breathing. If the patient is apneic, then a bag valve mask may be used or intubation performed. Antidote therapy is crucial and comprises naloxone, which may be given IV or IM in 5-10 mg bolus dosing followed by infusion. Intranasal formulation is also available. An eventual psychiatric evaluation is recommended for possible suicidality. 

## Conclusions

Our case reports acute cardiomyopathy and HFrEF resulting from recreational fentanyl intoxication in a young male without prior history of cardiac disease. Acute fentanyl intoxication is an emerging problem in the United States healthcare system; therefore, it is important to recognize such rare complications from it. POCUS may serve as a quick and convenient tool to screen for possible cardiomyopathy in such cases of opioid overdose where clinical signs and symptoms of acute heart failure are evident. Resuscitation should be prompt and based on standard principles of heart failure management along with the administration of antidote naloxone to prevent mortality.
